# Enteric bacterial agents associated with diarrhea and their antimicrobial sensitivity profiles in children under 5 years from mukuru informal settlement, Nairobi, Kenya

**DOI:** 10.1186/s12879-024-09114-5

**Published:** 2024-02-22

**Authors:** Susan Kiiru, John Maina, John Njeru Mwaniki, Edinah Songoro, Samuel Kariuki

**Affiliations:** 1 Center for Microbiology Research, Kenya Medical Research Institute, Nairobi, Kenya; 2 Jomo Kenyatta University of Agriculture and Technology, JKUAT, Juja, Kenya

**Keywords:** Bacteria enterics, Children under 5 years, Diarrhea, Antimicrobial resistance, Informal settlement

## Abstract

**Background:**

In Kenya, diarrhoeal disease is the third leading cause of child mortality after malaria and pneumonia, accounting for nearly 100 deaths daily. We conducted a cross-sectional study in Mukuru informal settlements to determine the bacteria associated with diarrhea and their ASTs to provide data essential for implementing appropriate intervention measures.

**Methods:**

Diarrheagenic children (≤ 5 years) were purposively recruited from outpatient clinics of Municipal City Council, Mukuru kwa Reuben, Medical Missionaries of Mary, and Mama Lucy Kibaki Hospital, Nairobi. A total of 219 stool samples were collected between May 2021 and August 2021. Stool culture was done on MacConkey and *Salmonella Shigella* agar, while the recovered bacteria were identified using VITEK®2GNID and polymerase chain reaction (PCR) used for *E. coli* pathotyping. Antibiotic Susceptibility Testing was done using VITEK®2AST-GN83.

**Results:**

At least one bacterial organism was recovered from each of the 213 (97%) participants, with 115 (56%) participants having only one bacterial type isolated, 90 (43%) with two types of bacteria, and 2 (1%) with three types of bacteria recovered. The most predominant bacteria recovered was 85% (93/109) non-pathogenic *E.coli* and 15% (16/109)of pathogenic *E.coli*, with 2 (1%) were Enterohemorrhagic *E.coli* (EHEC), 6 (3%) were Enteroaggregative *E.coli* (EAEC), and 8 (4%) were Enteropathogenic *E.coli* (EPEC). Other potentially pathogenic bacteria included *Enterobacter* sp (27.8%), Klebsiella sp 33(11%), and *Citrobacter* sp 15(4.7%). Pathogenic isolates such as *Salmonella 7 (*2%), *Proteus mirabilis* 16 (6%), *Providencia alcalifaciens 1 (*0.3%), and *Shigella* 16 (4.7%) were detected. Isolates such as *Pantoea* spp 2(0.67%), *Raoultella planticola* 1(0.33%), and *Kluyvera* 6(2%) rarely reported but implicated with opportunistic diarrhoeal disease were also recovered. Ampicillin, cefazolin, and sulfamethoxazole-trimethoprim were the least effective antimicrobials at 64%, 57%, and 55% resistance, respectively, while meropenem (99%), amikacin (99%), tazobactam piperacillin (96%), and cefepime (95%) were the most effective. Overall, 33(21%) of all enterics recovered were multidrug-resistant.

**Conclusion:**

The study documented different bacteria potentially implicated with childhood diarrhea that were not limited to *E. coli, Shigella, and Salmonella*, as previously observed in Kenya. The strains were resistant to the commonly used antibiotics, thus narrowing the treatment options for diarrheal disease.

## Background

Diarrhoeal disease is Kenya’s third leading cause of pediatric deaths after malaria and pneumonia [1]. According to UNICEF 2022, diarrhoeal diseases account for an estimated 1300 deaths per day in children below five years of age [2]. This burden is higher in informal settlements, characterized by insufficient toilet facilities, inadequate sewage drainage infrastructure, overcrowding, and poor sanitation practices. It is estimated that 48% of Kenya’s population lacks access to safe water and has limited sanitation amenities, resulting in improper disposal of fecal waste in the informal settlements [3, 4].

Although parasites and viruses have been associated with diarrhea diseases, bacteria are the most common aetiological agents, especially *Escherichia coli, Clostridium botulinum*, *Shigella, Staphylococcus aureus, Campylobacter jejuni, Vibrio cholera*, and *Salmonella* [5]. Children aged 5 years and below are at higher risk of foodborne bacterial infections caused by *Campylobacter jejuni, Vibrio cholerae, E.coli, Salmonella*,and*Shigella*species[6]. Approximately, Shigella causes about 125 million diarrhea episodes and 160,000 deaths every years, with a third are related to young children [7], while diarrheagenic E.coli causes 70,000 deaths in children under 5 years of age in Africa [8]. These bacteria are ingested along with unsafe food and water. They can also be contracted by touching animals suffering from diarrhea, polluted environments, or contact with a person’s stool. Acute diarrhea caused by these bacteria is often characterized by a massive loss of water that forms a large proportion of children’s body weight, leading to rapid dehydration that can lead to death. The severity of acute water loss is aggravated by children using more water daily due to their higher metabolic rates than adults [9].

The primary care for diarrhea involves the replacement of lost electrolytes and water through oral rehydration solution (ORS) [10]. The World Health Organization does not recommend antibiotic treatment of diarrhea in children, but traveler’s diarrhea, commonly associated with enterotoxigenic and enteroaggregative *E. coli*, non-typhoidal *Salmonella*, and *Shigella* spp, requires proper antibiotic therapy to shorten the bacterial excretion and manage the clinical symptoms [11, 12]. In our Kenyan settings, antibiotic treatment of diarrhea involves using oral metronidazole, azithromycin, ciprofloxacin, and amoxiclav [13]. The parenteral antibiotic intervention involves the use of ceftriaxone or ciprofloxacin injections [14] The continued use and misuse of these antimicrobials has resulted in high levels of resistance against commonly available antimicrobials. These enteric bacteria interact within the gut with the potential to transmit and exchange resistance genes, leading to the emergence of multidrug-resistant strains [15]. Diarrhea caused by multidrug-resistant bacteria can result in longer hospitalization, poor treatment outcomes, and increased mortality [16]. Despite drug-resistant enteric bacteria being a significant cause of children’s morbidity and mortality, the full extent of the burden of disease is not well known. This study sought to investigate the common enteric bacterial agents associated with childhood diarrhea and their corresponding antibiotic susceptibility patterns.

## Methodology

### Study design and site

A cross-sectional study design was employed to conduct the study between May 2021 and November 2021 in Mukuru informal settlement, the second-largest informal set-up in Kenya,. The participants were purposively recruited from outpatient clinics located in this informal settlement, in Municipal City Council (MCC), Mukuru kwa Reuben (MR), Medical Missionaries of Mary (MMM), and Mama Lucy Kibaki Hospital (MLKH) Nairobi, the referral facility for patients from MCC, MR, and MMM. MCC, MMM, and MR are level 1/ 2 facilities and thus do not have inpatient facilities, while MLKH operates as an outpatient and inpatient hospital with a 135-bed capacity and a 35-bed children’s ward on the ground floor of the inpatient wing. The ward is divided into an acute care and chronic care unit managing various medical conditions in children aged 1 month to 12 years of age. The clinics were selected because they have the capacity to handle over 1000 cases of diarrheal infections per month, they offer affordable services like a child welfare clinic, and the fact that the research team has long-standing professional relationships with the management of these hospitals.

### Study recruitment

A healthcare worker at each of the clinics identified patients presenting mainly with abdominal pain and intense and frequent urge for bowel movement, accompanied by other symptoms including vomiting, fever, dehydration, weight loss, and loose stool. Participants who had taken antibiotics within the previous 7 days to treat other illnesses were excluded from the study, as confirmed by the clinician and the guardian/parent of the child. The parents/guardians of the participants meeting the inclusion criteria were interviewed to obtain the history of their illness and then asked to provide a stool sample or a rectal swab. However, we did not recruit inpatient children and those who were being given parenteral antibiotics.

### Sampling

According to the Kenya Bureau of Statistics 2019 census data, 11.2% of Kenya’s population is between 0 and 4 years of age. Using these statistics, we estimated that from a total population of 150,000 people in Mukuru kwa Reuben and Njenga, 16, 800 of its population is below the age of 5 years (http://housingfinanceafrica.org/documents/2019-kenya-population-and-housing-census-reports/). A purposive sampling method (children presenting exclusively with diarrhea were recruited) was used to calculate the working sampling size from our target population of.

16, 800 children. Fisher’s sample size calculation method was used to establish the sample.

size in the study [17].

N = Z 2 P (l-P)/d 2.

Where N = Minimal sample size:

Z = Standard normal deviation corresponding to 95% confidence interval (= 1.96);

P = Estimated prevalence of diarrhoea in children under 5 in Kenya, which in this case is 15% [18].

d = degree of precision (5%).

= (1.96) 2 0.15(1-0.15)/ (0.05) 2 = 195.9216

*N* = 196 minimum fecal samples.

219 Stool samples per recruitment site were collected depending on how many children presented mainly with diarrhea and had met our inclusion criteria during the participants recruitment period.

### Stool sample collection

The fecal sample was collected in a poly pot by the guardian or the parent following guidance from the attending nurse. For children over two years, a cotton rectal swab was used and moistened to avoid discomfort to the child. The collected stool samples were labeled with unique identifiers, kept in Cary Blair transport media, transported to the Centre for Microbiology laboratory in a cool box, and processed within 24 h.

### Bacterial isolation and identification

Direct plating on MacConkey agar (Oxoid) and *Salmonella Shigella* agar (Oxoid)media was done in the lab. Enrichment was also done on Selenite Faecal broth(Oxoid) to optimize the recovery of *Salmonella* and incubated at 37 ℃ overnight [19]. The non-duplicate enteric colonies were identified using colony morphology, Gram staining, VITEK 2 system (bioMérieux), and PCR [20]. The following NTCT and ATCC organisms were used as positive controls for the detection of the enteric pathogens: NCTC 13,420 to help in the identification of *Acinetobacter baumannii*, NCTC 10,975 to identify *Proteus mirabilis*, NCTC 12,028 to identify *Morganella morganii*, NCTC 8900 to identify *Serratia marcescens*, *E.coli* ATCC 25,922 and *S. aureus* ATCC 25,923. For the recovered *E. coli isolates*, PCRs were done to identify pathotypes of *E.coli* associated with opportunistic pediatric diarrhea. The targeted genes for intestinal *E. coli* pathotypes included bundle-forming pilus(bfpA) of enteropathogenic *E.coli* (EPEC), Shiga toxin (stx1) of enterohemorrhagic *E.coli* (EHEC), transcriptional activator (aggR) of enteroaggregative *E.coli* (EAEC), heat-labile toxin (LT) of enterotoxigenic *E.coli* (ETEC), heat-stable toxin (sth) of ETEC, stp of ETEC, and afimbrial adhesins(afa) of diffusely adherent *E.coli* (DAEC) [21]. All *E. coli* isolates and commercially bought positive controls for each of the genes tested had their DNA extracted using the boiling method previously described by [22]. Briefly, a loopful of pure culture was emulsified in 1 ml distilled DNase/RNase-free water and boiled at 95 °C for 12 min in a heating block. Centrifuging for 5 min at 14 000 rotations per minute did the separation of bacterial cell debris and DNA. The extracted DNA’s quality and concentration were measured using 1% gel electrophoresis stained using SYBR green [23]. The DNA templates were amplified using specific oligonucleotide primer pairs, Table 1. Qiagen master mix was used, where the final volume was 25 µL in a master mix containing one µl forward primer(0.2µM), one µl reverse primer (0.2µM),11 µl PCR water,11 µl PCR mix (QIAGEN, Germany) with the PCR components such as; Taq DNA Polymerase(2.5units), PCR Buffer(1x), MgCl2 (0.2µM), and ultrapure dNTPs(200µM),) followed by addition of 1 µl template DNA. DNA amplification was performed using the following thermal profile for 35 cycles: initial denaturation for 5 min at 95 ℃, followed by final denaturation for 30 s at 94 ℃, variable annealing (shown in Table 1) for 1 min, initial extension for 2 min 30 s at 72 ℃ followed by final extension for 7 min at 72 ℃. 10 µl of amplicons were analyzed by electrophoresis on 1.5% agarose gel. The sizes of the amplicons were determined by comparing them with a 100 bp DNA ladder.

**Table 1:** *Escherichia coli* pathotypes genes analyzed, and the primers used

Genes	Primer sequence (5’-3’)	Product size Bp	Anneling temp
bfpA	F: AATGGTGCTTGCGCTTGCTGC	324	62
	R: CCGCTTTATCCAACCTGGTA		
stx1	F: AGTTAATGTGGTGGCGAA	817	58
	R:GACTTGCCGCTTCCAT		
aggR	F: GTATACACAAAGAAGGAAGC	254	58
	R: ACAGAATCGTCAGCATCAGC		
LT	F: GCACACGGAGCTCCTCAGTC	218	60
	R: TCCTTCATCCTTTCAATGGCTTT		
sth	F: CCCTCAGGATGCTAAACCAG	166	56
	R: TTAATAGCACCCGGTACAAGC		
stp	F: TCTGTATTATCTTTCCCCTC	186	65
	R: ATAACATCCAGCACAGGC		
afa	F: GCTGGGCAGCAAACTGATAACT	750	66
	R: CATCAAGCTGTTTGTTCGTCCGC		

### Antimicrobial susceptibility testing

Antibiotic susceptibility testing for 157 non-duplicate representative isolates of each genus was done using the VITEK 2®AST-GN83card on VITEK 2® automated platform. The minimum inhibitory concentration (MIC) interpretations were done using Clinical and Laboratory Standards Institute (CLSI 2022) and the VITEK 2 ® Advanced Expert System(AES), and the analysis using WHONET software followed. The following antimicrobial agents were tested: ampicillin (AMP), amoxicillin/clavulanic acid (AMC), ampicillin/ Sulbactam (SAM), piperacillin/tazobactam (TZP), cefazolin (CZO), cefuroxime(CXM), cefuroxime axetil (CXA), cefoxitin (FOX), cefotaxime (CTX), ceftazidime (CAZ), ceftriaxone(CRO), cefepime(FEP), aztreonam(ATM), meropenem (MEM), amikacin (AMK), gentamicin (GEN), ciprofloxacin (CIP, nitrofurantoin (NIT), and trimethoprim-sulfamethoxazole (SXT). The MICs generated were used to cluster the isolates into various resistance profiles ranging from fully sensitive, intermediate, and resistant. Isolates exhibiting resistance to 3 or more classes or subclasses of antibiotics were scored as Multidrug-resistant (MDR) as defined by the European Center for Disease Control (ECDC) [24], while those that were resistant to penicillins, 1st,2nd, 3rd cephalosporins and aztreonam were scored as ESBLs(Extended Spectrum Beta-lactamase). Isolates, both intermediate and resistant to meropenem, were scored as carbapenemases, while isolates with combined resistance to β-lactams, fluoroquinolone, and aminoglycosides such as gentamicin were denoted as βFQA-strains [24].

### Data analysis

Data was analyzed using the Statistical Package for Social Sciences (SPSS), version 20. Descriptive analysis was used to show the distribution of recovered bacterial enteric per recruitment site, gender, and age group. Antimicrobial-resistant patterns were interpreted and analysed using the M 100 Clinical and Laboratory Standards Institute (CLSI 2022) and the VITEK 2® Advanced Expert System (AES). And the Antimicrobial resistant patters were analyzed using WHONET 2023 https://whonet.org/. Moreover the statistical significance of distribution of recovered bacteria isolates between gender, age group and recruitment site was done using Fisher’s exact test.

## Results

### Patients’ demographic characteristics

Of the 219 participants recruited, 117(53%) were male. The participants in age category 12–24 were significantly higher than other age categories, *p* < 0.001, as shown in Table 2.

**Table 2:** demographic characteristics of the patients recruited in four recruitment sites

Demographic characterists		Recruitment site				Total (*N* = 219)	X² *P*-Value
			Health centres		Level 4		
		MR (*N* = 32)	MMM (*N* = 112)	MCC (*N* = 42)	MLK (*N* = 33)		
Gender	Female	18(56)	48(42)	22(52)	13(42)	101(47)	0.459
Age_Category(in months)	0–11	11(34)	26(23)	6(14)	0	42(19)	< 0.001
	12- 24	13(40)	31(28)	5(12)	5(15)	54(25)	
	25–36	4(13)	18(16)	12(29)	3(9)	37(17)	
	37–48	1(3)	25(22)	8(19)	8(24)	42(19)	
	49–60	3(9)	12(11)	11(26)	17(52)	43(20)	

Key: MR-Mukuru kwa Reuben, MMM-Medical Missionaries of Mary, MLK-Mama Lucy Kibaki Hospital, and MCC- Municipal City Council. Numbers in brackets represent %

### Bacterial isolation

**Fig. 1:**
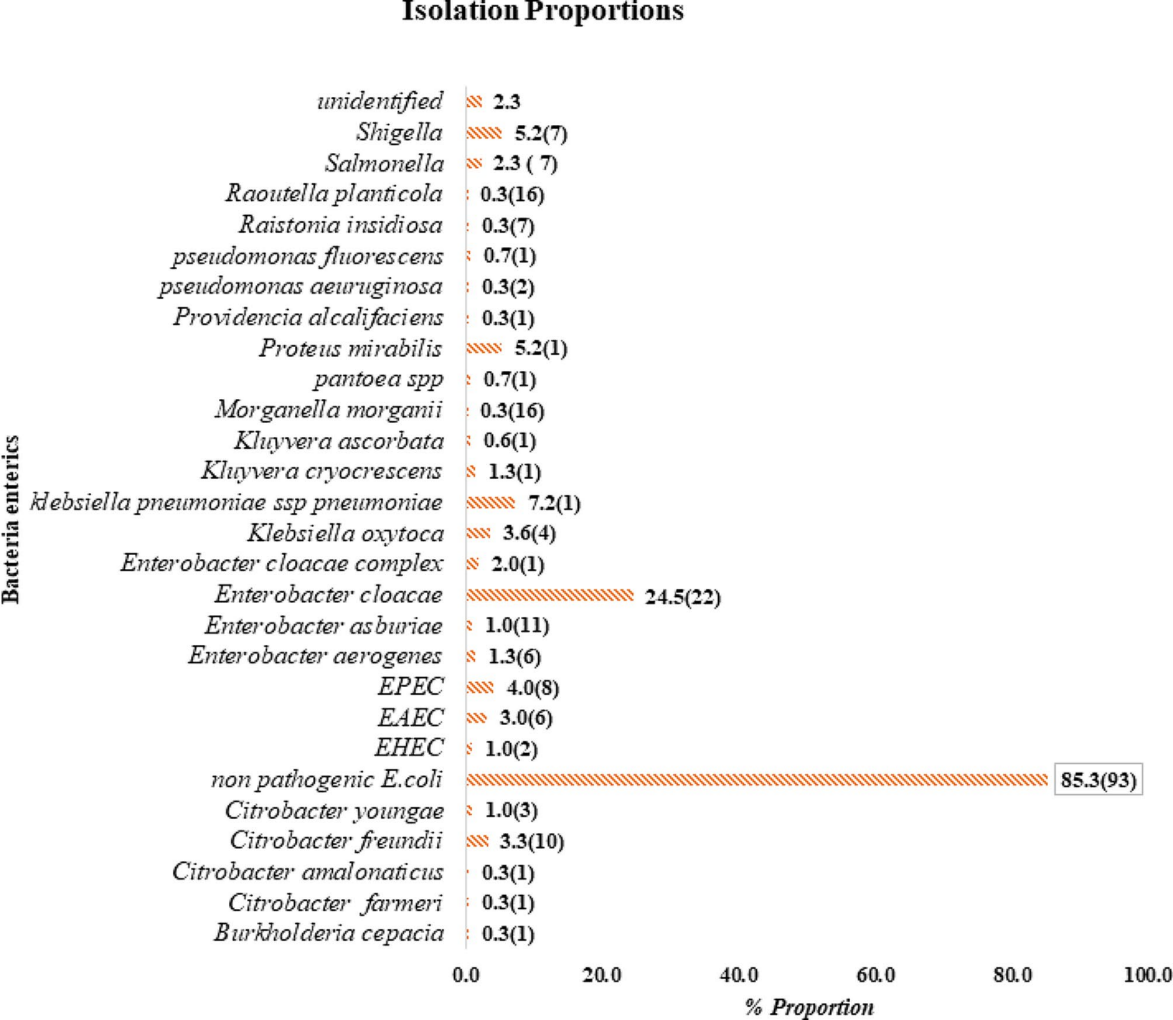
percentage proportion of bacterial enterics isolated. (n) represents the number of enterics recovered. Enteropathogenic *E.coli* (EPEC), Enterohemorrhagic *E.coli* (EHEC), and Enteroaggregative *E.coli* (EAEC)

At least one bacterial organism was recovered from 213 (97%) participants, with 115(56%) participants having only one bacterial type isolated and 43.2% (92/213) polyculture, with 90(43%) with two types of bacteria recovered and the common combination being *Enterobacter cloacae* and *E.coli*, and 2(1%) with three types of bacteria recovered (*Shigella*, *Proteus mirabilis* and *Enterobacter cloacae* combination while the other combination being *Salmonella*, *Proteus mirabilis* and *Klebsiella Pnuemoniae)*. The most predominant bacteria recovered was 85%(93/109) non-pathogenic *E.coli* and 15% (16/109)of pathogenic *E.coli*, with 2 (1%) were Enterohemorrhagic *E.coli* (EHEC), 6 (3%) were Enteroaggregative *E.coli* (EAEC), and 8 (4%) were Enteropathogenic *E.coli*(EPEC). Other potentially pathogenic bacteria included *Enterobacter* sp (27.8%), *Klebsiella* sp 33(11%), and *Citrobacter* sp 15(4.7%), with specific species of each genus shown in Fig. 1. Other pathogenic isolates such as *Salmonella* spp 7 (2%), *Proteus mirabilis* 16 (6%), *Providencia alcalifaciens* 1 (0.3%), and *Shigella* spp 16 (4.7%) were also isolated. Rarely reported but implicated with opportunistic diarrhoeal disease diseases isolates such as *Pantoea* spp 2 (0.67%), *Raoultella planticola* 1(0.33%), and *Kluyvera spp* 5 (2) were also recovered, as depicted in Fig. 1 below.

### Antibiotic resistance patterns

**Fig. 2:**
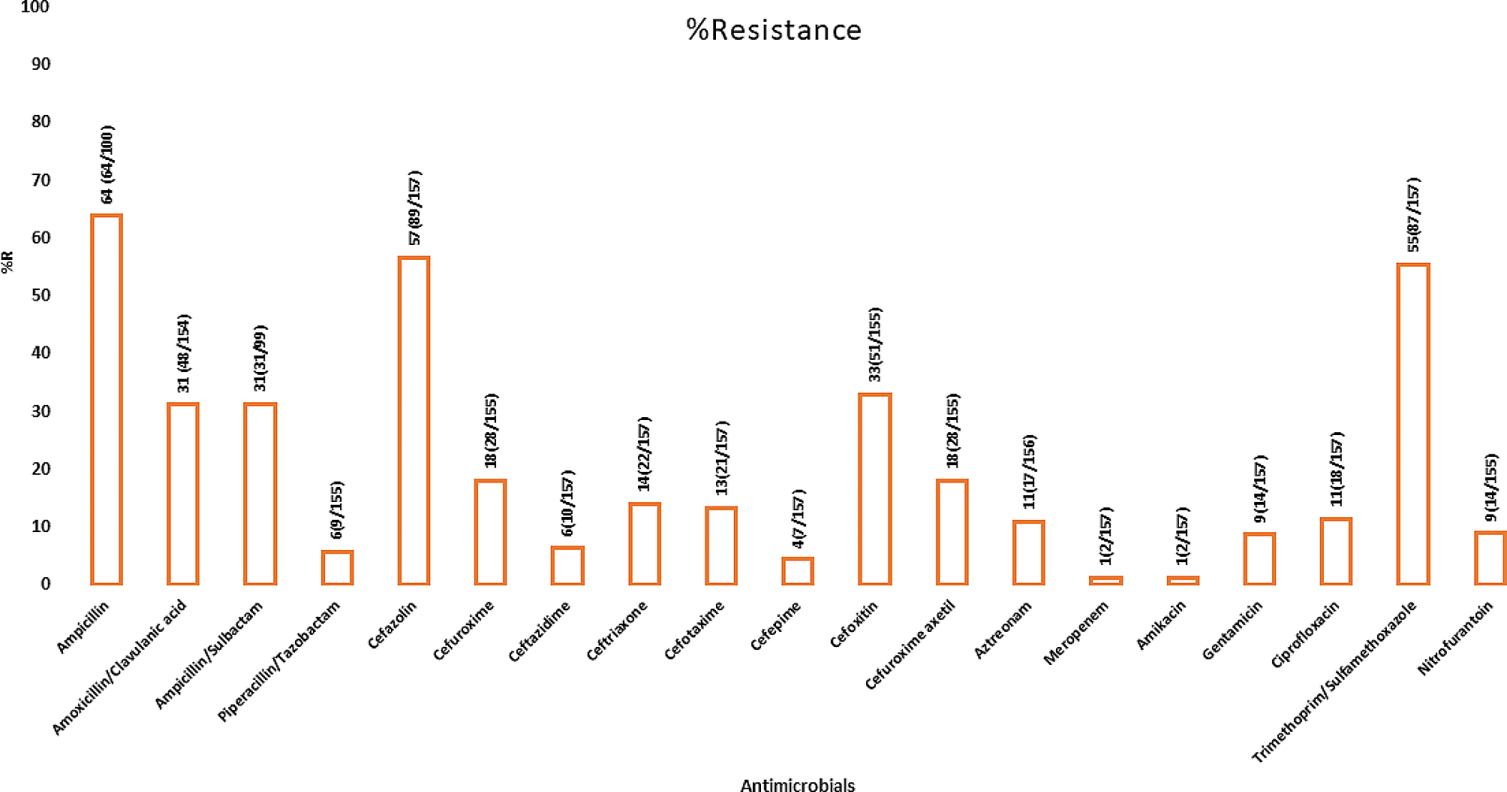
Antibiotic-resistant patterns (%) for the recovered bacteria enterics. (n/157) represents the number of bacteria enterics resistant to the antimicrobials out of 157 bacteria subjected to AST. * ampicillin and ampicillin sulbactam resistance were out 100 and 99, respectively, since the Vitek 2 VITEK 2® automated platform did not test it against organisms with intrinsic resistance, including *Citrobacter freundii*, *Enterobacter cloacae*, *Enterobacter aerogenes*, and *Morganella morganii*

Overall, ampicillin (AMP), sulfamethoxazole-trimethoprim (STX), and cefazolin (CZO) were the least effective antimicrobials, with resistance at 64%, 54% & 51%, respectively. Similarly, relatively high resistance towards cefoxitin (33%) and ampicillin-sulbactam (31%) was also recorded. Meropenem, amikacin, tazobactam piperacillin, and cefepime were the most effective, with less than 5% resistance. For antibiotics commonly used for the treatment of diarrhea resistance levels were; amoxiclav (31%), ceftriaxone (14%), and ciprofloxacin (11%), Fig. 2. The resistance levels towards ceftazidime, ceftriaxone, and cefepime range between 5 and 14%. Among antibiotic-inhibitor combinations, amoxicillin-clavulanic acid and ampicillin-sulbactam resistance were at 31%, while for tazobactam piperacillin resistance was (4%).

Genus-specific analysis showed that *E coli* isolates were resistant to at least 1 of the 20 antibiotics tested, and two (3.6%) non-pathogenic *E. coli* isolates were resistant to meropenem, as shown in Table 3. *E.coli, Enterobacter* spp & *Klebsiella* spp were the most resistant to broad-spectrum antimicrobials such as ceftazidime, ceftriaxone ciprofloxacin, gentamicin, and cefepime, ranging between 6 and 33%. Resistance to all 20 antibiotics was high in the age group 12–24 months, while isolates from participants 0–11 months were the least resistant. Notably, meropenem resistance in the non-pathogenic *E.coli* was recorded in two recruitment sites, MMM and MCC. Gentamicin resistance was noted in isolates recovered from the MMM (7.4%), MCC (14.3%), and MR (15%) and not in the referral hospital MLK, while amikacin resistance was only recorded in MLK (4.8%) hospital. The isolates recovered as polyculture slightly differed in antibiotic susceptibility patterns for all tested antibiotics. For instance, one patient had *Klebsiella pneumoniae*, and *E.coli* recovered from their stool. Their ASTs patterns differed, where *Klebsiella pneumoniae* was fully susceptible to aztreonam, cefepime, and piperacillin-tazobactam, but *E.coli* showed resistance ranging between 1 and 6%.

**Table 3:** Antimicrobial resistance profile across the genera of the recovered bacterial enterics. (*n*) represents the number of bacteria enterics resistant to the antimicrobial

Genus		Antimicrobial resistance profiles: *n* (%)																		
	*n*	AMP	AMC	SAM	TZP	CFZ	CXM	CAZ	CRO	CTX	FEP	FOX	CAE	ATM	MEM	AMK	GEN	CIP	SMX	NIT
Citrobacter spp	10		1(10)		0	9(81.8)	0	0	0	0	0	2(20)	0	0	0	0	1(9.1)	1(9.1)	3(27.3)	0
***E.coli***	**56**	**39(72)**	**7(13)**	**24(44)**	**8(15)**	**28(50)**	**17(30)**	**8(14)**	**17(30)**	**16(27)**	**6(11)**	**7(13)**	**17(30)**	**25(14)**	**2(4)**	**1(1.8)**	**7(13)**	**10(18)**	**41(73)**	**2(4)**
***Enterobacter spp***	**38**	**1(3)**	**27(71)**		**1(3)**	**32(84)**	**2(5)**	**0**	**1(3)**	**1(3)**	**0**	**30(79)**	**2(5)**	**1(3)**	**0**	**0**	**2(5)**	**2(5)**	**11(29)**	**1(3)**
***Klebsiella spp***	**21**	**13(62)**	**4(19)**	**3(15)**	**0**	**6(29)**	**5(23)**	**1(5)**	**3(15)**	**3(15)**	**0**	**5(23)**	**5(23)**	**1(5)**	**0**	**0**	**2(10)**	**2(10)**	**10(48)**	**1(5)**
***Kluyviella spp***	**4**	**1(25)**	**0**	**0**	**0**	**3(75)**	**1(25)**	**1(25)**	**1(25)**	**1(25)**	**1(25)**	**1(25)**	**1(25)**	**1(25)**	**0**	**1(25)**	**1(25)**	**1(25)**	**4(100)**	**0**
***Morgenella spp***	**1**	**1(100)**	**1(100)**	**1(100)**	**0**	**1(100)**	**1(100)**	**0**	**0**	**0**	**0**	**0**	**1(100)**	**0**	**0**	**0**	**0**	**0**	**1(100)**	**0**
***Proteus spp***	**13**	**5(46)**	**3(23)**	**0**	**0**	**4(31)**	**1(8)**	**0**	**0**	**0**	**0**	**3(23)**	**1(8)**	**0**	**0**	**0**	**0**	**1(8)**	**8(62)**	**7(54)**
***Providencia spp***	**1**	**0**		**0**	**0**	**0**	**0**	**0**	**0**	**0**	**0**	**0**	**0**	**0**	**0**	**0**	**0**	**0**	**1(100)**	**1(100)**
***Pseudomonas spp***	**2**					**0**		**0**	**0**	**0**	**0**				**0**	**0**	**0**	**0**	**0**	
***Raoutella spp***	**1**	**0**	**0**	**0**	**0**	**0**	**0**	**0**	**0**	**0**	**0**	**0**	**0**	**0**	**0**	**0**	**0**	**0**	**1(100)**	**0**
***Salmonella spp***	**3**	**3(100)**	**1(33)**	**1(33)**	**0**	**2(66)**	**0**	**0**	**0**	**0**	**0**	**1(33)**	**0**	**0**	**0**	**0**	**1(33)**	**1(33)**	**3(100)**	**0**
***Shigella spp***	**5**	**2(40)**	**3(60)**	**2(40)**	**0**	**3(60)**	**1(20)**	**0**	**0**	**0**	**0**	**1(20)**	**1(20)**	**0**	**0**	**0**	**0**	**0**	**3(60)**	**1(20)**
***pantoea spp***	**1**		**1(100)**		**0**	**1(100)**	**0**	**0**	**0**	**0**	**0**	**1(100)**	**0**	**0**	**0**	**0**	**0**	**0**	**1(100)**	**1(100)**

Key: AMP- ampicillin, AMC- amoxicillin clavulanic acid, SAM- ampicillin sulbactam, TZP- tazobactam piperacillin, CZO- cefazolin, CXM- cefuroxime, CAZ- ceftazidime, CRO- ceftriaxone, CTX- cefotaxime, FEP- cefepime, FOX- cefoxitin, CAE- cefuroxime axetil, ATM- aztreonam, MEM- meropenem, AMK- amikacin, GEN- gentamicin, CIP- ciprofloxacin, SMX- sulfamethoxazole-trimethoprim, NIT- nitrofurantoin. *E.coli- Escherichia coli*, spp represents species, (_) indicate drug not tested, n represents the percentage resistance.

**Fig. 3:**
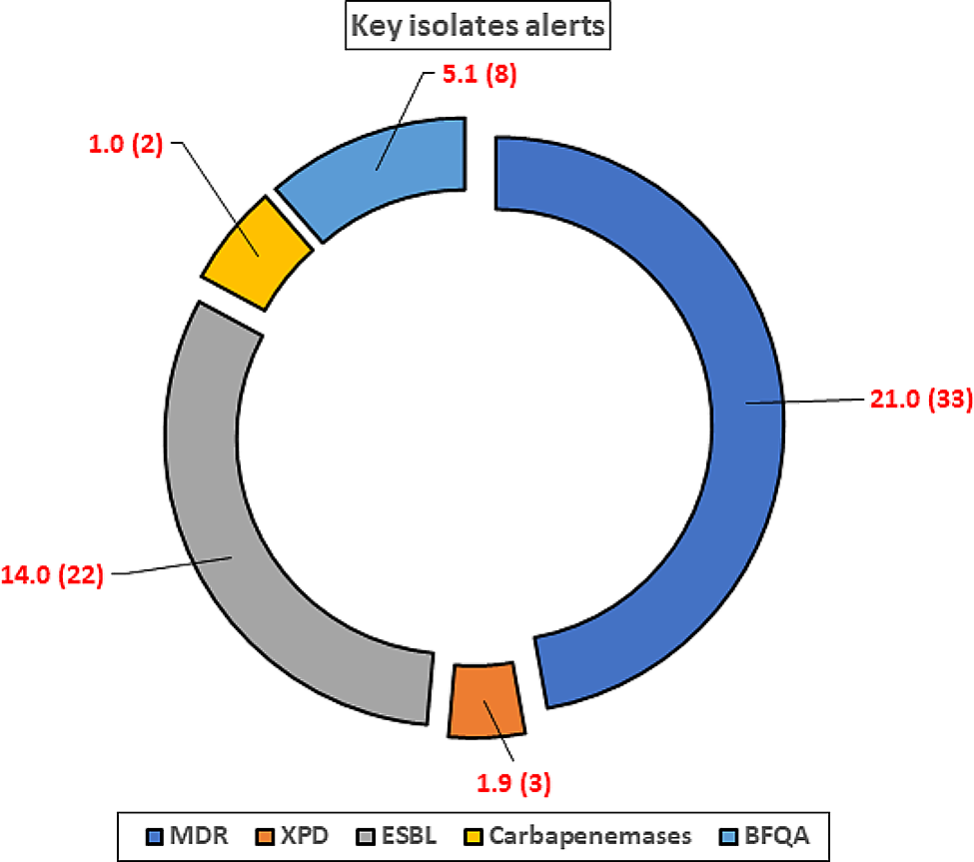
Important resistance alerts portrayed by the recovered bacterial enterics. () represents the actual number of isolates. MDR- multidrug-resistant isolates, ESBL-expanded spectrum beta lactamases, XPD/XDR-Extensively drug-resistant isolates, BFQA- Beta-lactam, Fluoro/Quinolones, Aminoglycosides

A total of 33(21%) bacterial isolates were multidrug-resistant (MDR) organisms resistant to three or more classes of antibiotics, with the most common MDR phenotype being a combination of ampicillin, amoxiclav, ampicillin-sulbactam, aztreonam, cefazolin, and sulfamethoxazole-trimethoprim at 13%. Of the 33 MDR isolates, 26 (78.8%) were *E.coli*, with 2 EPEC and 1 EAEC, 2(6%) were *Klebsiella pneumoniae*, 1(3%) were *Klebsiella oxytoca*, 1(3%) *Proteus mirabillis*,1(3%) *Kluyvera cryoscrescence* and 2(6%) was *Enterobacter cloacae.* Moreover, 3(1.9%) non-pathogenic *E.coli*, each from MMM, MCC, and MR, were extensively drug-resistant (XDR) to 6/7 classes of antibiotics tested. A total of 22(14%) were Expanded Spectrum Beta Lactamase (ESBL) producers, presented by 18 *E.coli*, 1 Klebsiella oxytoca, 1 Klebsiella pneumoniae, 1 *Kluyvera cryoscrescence* and 1 *Enterobacter cloacae* from all the recruitment sites, while 2(1%) non-pathogenic *E.coli* were carbapenemase producers from MMM and MLK, and 8(5%),7 E.*coli* and1 *Klebsiella pneumoniae* from the 4 hospitals had Beta-lactam, Fluoro/Quinolones, Aminoglycosides(BFQA) resistance phenotype, as shown in Fig. 3.

## Discussion

The recovery of at least one bacterial enteric from 213/219(97%) implies bacterial etiologies are a significant cause of diarrhea illnesses in children under five years. 43.2% (92/213) of participants were polyculture, with the recovery of the normal intestinal flora alongside pathogenic bacteria enterics associated with childhood diarrhea. The most dominant pathogenic *E.coli* isolation rate at 15% % (16/109), with 1% EHEC, 4% EPEC, and 3% EAEC, confirms *E.coli* as a leading cause of diarrhea in under-five years child in Mukuru. Our finding differed from the Zelelie et al. study conducted in Ethiopia among children under five years of age with acute diarrhea and hospitalized or visited the institutions as an outpatient, where the EPEC isolation rate was 13%, 6% EHEC, and 6% EAEC [25]. Moreover, this study’s findings contrast Webale et al. (2020) study conducted in children under five years visiting Mbagathi Hospital, where 36.4% wre pathogenic *E.coli*, with 20.9% EAEC, 10.2% Enterotoxigenic *E.coli* (ETEC), 4% EPEC, and 0.5% Enteroinvasive *E.coli* (EIEC) [26]. Similarly, a study by Impwi et al. (2016) carried out in Mukuru informal settlement in diarrheagenic children under five years had a different isolation rate of *E.coli*, where 27.1% was EAEC, 37.4% EPEC, 27.1% ETEC, 4.2% EHEC,and 4.2% EIEC [27].In the neighboring Uganda, the isolation rate of EPEC was 6.7%, 14.2% EAC, and 3% EAEC, from a study conducted among inpatient children under 5 years [28], disagreeing with our findings. The varying results across different geographical locations and regions could imply pathogenic *E.coli* burden varies with locations. The variations could also have been attributed to different inclusion criteria in the study, for instance, Zelelie et al.’s study recruited children who were interacting with calves, while the study conducted in Uganda had inpatient children only. This suggests a need for standardized testing worldwide for diarrhea in children for better surveillance and comparison in the future.

The study also identified rarely reported isolates but implicated with diarrhea diseases such as *Pantoea* spp, *Raoultella planticola*, and *Kluyvera*, while other previous studies in Kenya did not [15, 17, 29]. The current study used VITEK 2®system (bioMérieux) for comprehensive bacterial identification, while these previous studies used the basic biochemical and serotyping methods, signifying a more sensitive automated identification method is superior and should be adopted for robust identification and better treatment plans. Our findings had a lower *Pantoea* spp isolate rate than a cross-sectional study conducted in two Nigerian healthcare centers among infants with 3.85% [30]. The *Raoultella planticola* isolation rate in the current study was lower than Afum’s and colleagues’ cross-sectional study conducted among diarrheagenic patients in Ghana, with a 2% isolaion rate [31]. The variations in the isolation rate could be attributed to the burden varying with locations.

The study identified *Enterobacter* spp as the second dominant bacterial enterics. Among them, *Enterobacter cloacae* was the most dominant(24.5%). These findings differ from previous studies, which identified *Shigella* spp as the second most dominant [26, 27, 29]. Usually, *Enterobacter cloacae*, together with *Citrobacter freundii*, *Enterobacter cloacae complex*, *Enterobacter aerogenes*, and *Morganella morganii*, are a gut-normal flora that becomes pathogenic when the bacterial load increases, and they acquire virulence genes from other pathogenic isolates. Our study, however, had a 5.2% *Shigella* isolation rate, a lower rate than 83.8% from a study conducted in Kenya during two different climatic seasons [32]. The differences in the isolation rates could imply changes in trends of bacteria associated with childhood diarrhea. Other possibilities could be related to the study’s comprehensive identification system, recruitment of patients with only acute bloody diarrhea, and climatic seasons aspects in the inclusion criteria.

The study recovered *Proteus mirabilis* among the bacterial associated with diarrhoea in children under five years. The isolation rate(5.2%) was inconsistent with a 14.5% rate from a study conducted in the same informal settlement four years ago from the day of sample collection [27]. These findings agree with a study conducted in Accra, Ghana, where *Proteus mirabilis* was isolated at 5.7%, slightly higher than our study’s rate of 5.2% [33]Thse findings imply that *Proteus mirabilis* is a common cause of childhood diarrhea, which also agrees with findings from Yu et al. (2015). The isolation rate of Salmonella spp (2.3%) agreed with the 2.4% isolation rate from similar studies in Mbagathi Hospital [26].. However, this increased from 1.5% in 2016 [26], suggesting changes in patterns of bacteria in circulation in the informal settlement.

A significant proportion of the recovered bacterial enterics were resistant to commonly used antibiotics to manage diarrhea. There was 64% resistance to ampicillin, 55% to sulfamethoxazole-trimethoprim, and 57% to cefazolin. It is important to note that some of the recovered bacteria, such as *Citrobacter freundii*, *Enterobacter cloacae*, *Enterobacter aerogenes*, and *Morganella morganii*, contain intrinsic resistance to ampicillin [34] hence the VITEK 2®system (bioMérieux) did not test for it. The high resistance to AMP, SXT (Septrin ™), and CZO could be attributed to the fact that they are readily available in informal settings as over-the-counter drugs [35]. Our findings are consistent with Impwi et al. (2016), which documented high resistance to ampicillin and sulfamethoxazole-trimethoprim [27]. This implies that the resistance levels have been constant in these settings, and there should be a change in treatment options available for better management of bacterial infections.

Additionally, the study documented a 33% resistance toward cefoxitin, differing from Impwi et al. (2016) study conducted in the Mukuru informal settlement, showing a 64% resistance [27]. This variation could have resulted from the recruitment differences where Impwi and colleagues recruited children under five without diarrhea while our study recruited diarrheagenic pediatrics. Moreover, the four-year difference could have allowed for more antimicrobial stewardship in the settings. The high-level resistance to cefoxitin could be associated with the intrinsic resistance of some of the recovered enterics, such as *Citrobacter freundii*, *Enterobacter cloacae*, and *Enterobacter aerogenes* [34].

There was 1% carbapenem resistance for non-pathogenic *E.coli*. These findings are slightly lower than Dela et al. (2022) findings that documented a 4% meropenem resistance by *E.coli* recovered from diarrheagenic patients in Accra, Ghana [36]. Although the differences in the recruitment criteria and the testing methods applied in the studies could have accounted for the variations in resistance, the results imply that resistance varies across geographical locations and regions. The findings are worrying as commensal *E. coli* is harboring carbapenem resistance, and horizontal gene transfer can spread resistance to other pathogenic microorganisms, resulting in severe bacterial infections with reduced treatment options. Additionally, resistance to aminoglycosides would signify differences in antibiotics used in hospitals, as amikacin is majorly given as an injection, and this could imply that resistance recorded in MLKH, a referral hospital, uses it. Also, co-resistance between beta-lactams, fluoro/quinolones, and aminoglycosides suggests limited treatment options for childhood diarrhoea, with severe consequences, including increased mortality rates.

### Study limitations

The data was collected from children that had visited the clinics to seek treatments for diarrhoea; therefore, a true community prevalence of diarrhoea in children in the Mukuru informal settlement could not be reflected. However, these groups form an important part that can be used to extrapolate what happens in the community, as they are commonly treated with readily available antibiotics. The study was also conducted immediately after the COVID-19 lockdowns, which limited the sample size as people were still hesitant to visit the hospitals. Due to logistical constraints, only 157 samples were subjected to Antimicrobial Susceptibility Testing, and only *E.coli* isolates had their pathogenicity confirmed.

## Conclusion

The study documented different bacteria implicated as aetiologies of childhood diarrhoea that were not limited to *E.coli, Shigella*, and *Salmonella*, as previously observed in Kenya. The presence of Meropenem-resistant non-pathogenic *E.coli* suggests the possible emergence of carbapenem-resistant strains in the Mukuru informal settlement. Recovery of MDR strains suggests emerging limited treatment options for childhood diarrhoea, with severe consequences, including increased mortality rates. These findings, therefore, indicate the need for more robust standardized surveillance to confirm the patterns observed. Increasing resistance towards amoxiclav implies narrowed treatment options for diarrhoea, posing a public health threat.

## Data Availability

The data that support the findings of this study are available from the corresponding author, but restrictions apply to the availability of these data, which were used under license for the current study, and so are not publicly available. Data are, however, available from the authors upon reasonable request and with permission of NACOSTI.

## References

[1] Khan DSA, Naseem R, Salam RA, Lassi ZS, Das JK, Bhutta ZA. Interventions for high-burden infectious diseases in Children and adolescents: a Meta-analysis. Pediatrics 2022;149. 10.1542/PEDS.2021-053852C. 10.1542/peds.2021-053852C 35503332

[2] UNICEF. Diarrhoea - UNICEF DATA. UNICEF Official Page; 2022.

[3] Mbae CK, Nokes DJ, Mulinge E, Nyambura J, Waruru A, Kariuki S (2013). Intestinal parasitic infections in children presenting with diarrhoea in outpatient and inpatient settings in an informal settlement of Nairobi, Kenya. BMC Infect Dis.

[4] A System of Health Accounts. A System of Health Accounts 2011. 10.1787/9789264116016-EN.

[5] Zhang SX, Zhou YM, Tian LG, Chen JX, Tinoco-Torres R, Serrano E et al. Antibiotic resistance and molecular characterization of diarrheagenic Escherichia coli and non-typhoidal Salmonella strains isolated from infections in Southwest China. Infect Dis Poverty 2018;7. 10.1186/S40249-018-0427-2/ASSET/1FF4DD78-E272-4B0C-A564-691EC19B0B40/ASSETS/GRAPHIC/2095-5162-07-03-103-F002.PNG. 10.1186/s40249-018-0427-2 PMC5964730 29792233

[6] Moharana SS, Panda RK, Dash M, Chayani N, Bokade P, Pati S (2019). Etiology of childhood diarrhoea among under five children and molecular analysis of antibiotic resistance in isolated enteric bacterial pathogens from a tertiary care hospital, eastern odisha, India. BMC Infect Dis.

[7] Baker S, The HC. Recent insights into Shigella: a major contributor to the global diarrhoeal disease burden. Curr Opin Infect Dis. 2018;31. 10.1097/QCO.0000000000000475. 10.1097/QCO.0000000000000475 PMC6143181 30048255

[8] WHO. World Health organization. The top 10 causes of death Available from: https://www.who.int/news-room/fact-sheets/detail/the-top-10-causes-ofdeath. Https://WwwWhoInt/News-Room/Fact-Sheets/Detail/the-Top-10-Causes-of-Death 2020.

[9] Jéquier E, Constant F (2009). Water as an essential nutrient: the physiological basis of hydration. Eur J Clin Nutr 2010.

[10] Grenov B, Lanyero B, Nabukeera-Barungi N, Namusoke H, Ritz C, Friis H, et al. Diarrhea, dehydration, and the Associated Mortality in children with complicated severe Acute Malnutrition: a prospective cohort study in Uganda. J Pediatr. 2019;210. 10.1016/j.jpeds.2019.03.014. 10.1016/j.jpeds.2019.03.014 30992218

[11] World Health Organization. Technical updates of the guidelines on the Integrated Management of Childhood Illness (IMCI). Production 2005.

[12] Tribble DR. Antibiotic therapy for acute watery diarrhea and dysentery. Mil Med. 2017;182. 10.7205/MILMED-D-17-00068. 10.7205/MILMED-D-17-00068 PMC5650106 28885920

[13] Rhee C, Aol G, Ouma A, Audi A, Muema S, Auko J et al. Inappropriate use of antibiotics for childhood diarrhea case management - Kenya, 2009–2016. BMC Public Health 2019;19. 10.1186/s12889-019-6771-8. 10.1186/s12889-019-6771-8 PMC6696675 32326936

[14] Guarino A, Bruzzese E, Giannattasio A. Antibiotic treatment of acute gastroenteritis in children. F1000Res. 2018;7. 10.12688/F1000RESEARCH.12328.1/DOI. 10.12688/f1000research.12328.1 PMC5814741 29511533

[15] Tang Y, Fang L, Xu C, Zhang Q (2017). Antibiotic resistance trends and mechanisms in the foodborne pathogen, Campylobacter. Anim Health Res Rev.

[16] Neupane R, Bhathena M, Das G, Long E, Beard J, Solomon H, et al. Antibiotic resistance trends for common bacterial aetiologies of childhood diarrhoea in low- and middle-income countries: a systematic review. J Glob Health. 2023;13. 10.7189/JOGH.13.04060. 10.7189/jogh.13.04060 PMC10359834 37475599

[17] Jung SH. Stratified Fisher’s exact test and its sample size calculation. Biom J. 2014;56. 10.1002/bimj.201300048. 10.1002/bimj.201300048 PMC3884832 24395208

[18] Samuel SK, Moses NM, Emily TJ (2019). Prevalence of Enterobacteriaceae isolated from Childhood Diarrhoea in Mukuru Slums, Nairobi- Kenya. J Adv Microbiol.

[19] de Boer MD, de Boer RF, Lameijer A, Sterne E, Skidmore B, Suijkerbuijk AWM et al. Selenite enrichment broth to improve the sensitivity in molecular diagnostics of Salmonella. J Microbiol Methods 2019;157. 10.1016/j.mimet.2018.12.018. 10.1016/j.mimet.2018.12.018 30586562

[20] Kohlerschmidt DJ, Mingle LA, Dumas NB, Nattanmai G (2021). Identification of aerobic gram-negative Bacteria. Practical Handb Microbiol.

[21] Kademane A, Dixit M, Vasundhara. A Comprehensive Review of the Pathogenesis and virulence factors of E. Coli. Salud Ciencia Y Tecnologia. 2023;3. 10.56294/saludcyt2023411.

[22] Abdelhai H. M. Comparative study of Rapid DNA extraction methods of pathogenic Bacteria. Am J Bioscience Bioeng 2016;4. 10.11648/j.bio.20160401.11.

[23] Pooniya S, Lalwani S, Raina A, Millo T, Dogra T, Das. Quality and quantity of extracted deoxyribonucleic acid (DNA) from preserved soft tissues of Putrefied Unidentifiable Human Corpse. J Lab Physicians. 2014;6. 10.4103/0974-2727.129088. 10.4103/0974-2727.129088 PMC3969639 24696558

[24] Magiorakos AP, Srinivasan A, Carey RB, Carmeli Y, Falagas ME, Giske CG, et al. Multidrug-resistant, extensively drug-resistant and pandrug-resistant bacteria: an international expert proposal for interim standard definitions for acquired resistance. Clin Microbiol Infect. 2012;18. 10.1111/j.1469-0691.2011.03570.x. 10.1111/j.1469-0691.2011.03570.x 21793988

[25] Zelelie TZ, Eguale T, Yitayew B, Abeje D, Alemu A, Seman A, et al. Molecular epidemiology and antimicrobial susceptibility of diarrheagenic Escherichia coli isolated from children under age five with and without diarrhea in Central Ethiopia. PLoS ONE. 2023;18. 10.1371/journal.pone.0288517. 10.1371/journal.pone.0288517 PMC10348587 37450423

[26] Webale MK, Wanjala C, Guyah B, Shaviya N, Munyekenye GO, Nyanga PL et al. Epidemiological patterns and antimicrobial resistance of bacterial diarrhea among children in Nairobi City, Kenya. Gastroenterol Hepatol Bed Bench 2020;13. 10.22037/ghfbb.v13i3.1910. PMC7417493 32821354

[27] Impwi PM, Wambugu P, Kimang’a AN, Kariuki S. Pathogenic e.Coli and other pathogenic gram negative enteric strains from foecal samples of children without diarrhoea living in mukuru slums, Nairobi. East Afr Med J 2016;93.

[28] Masiga F, Kigozi E, Najjuka CF, Kajumbula H, Kateete DP. Diarrhoeagenic Escherichia coli isolated from children with acute diarrhoea at Rakai hospital, Southern Uganda. Afr Health Sci 2022;22. 10.4314/ahs.v22i1.67. 10.4314/ahs.v22i1.67 PMC9382479 36032447

[29] Olipher M, Johnstone M, Anne M, Joseph M, Tom M, Mwau M (2020). Antibacterial spectrum and susceptibility of bacterial pathogens causing Diarrheal illnesses: Cross Sectional Study of Patients Visiting Health Facility in Lake Victoria Region - Kenya. East Afr Sci.

[30] Pius Okiki A, Pius OA, Olubunmi OC. Medicine and Nursing www.iiste.orgISSN.vol.19.2015.

[31] Afum T, Asandem DA, Asare P, Asante-Poku A, Mensah GI, Musah AB, et al. Diarrhea-causing Bacteria and their antibiotic resistance patterns among Diarrhea patients from Ghana. Front Microbiol. 2022;13. 10.3389/fmicb.2022.894319. 10.3389/fmicb.2022.894319 PMC9161929 35663873

[32] Njuguna C, Njeru I, Mgamb E, Langat D, Makokha A, Ongore D (2016). Enteric pathogens and factors associated with acute bloody diarrhoea, Kenya. BMC Infect Dis.

[33] Dela H, Egyir B, Majekodunmi AO, Behene E, Yeboah C, Ackah D (2022). Diarrhoeagenic E. Coli occurrence and antimicrobial resistance of Extended Spectrum Beta-Lactamases isolated from diarrhoea patients attending health facilities in Accra, Ghana. PLoS ONE.

[34] Magiorakos A-P, Srinivasan A, Carey RB, Carmeli Y, Falagas ME, Giske CG et al. Multidrug-resistant, extensively drug-resistant and pandrug-resistant bacteria: an international expert proposal for interim standard definitions for acquired resistance background emergence of resistance to multiple antimicrobial agents in pathogenic bac. Clin Microbiol Infect 2012;18. 10.1111/j.1469-0691.2011.03570.x 21793988

[35] Mukokinya MA, Opanga S, Oluka M, Godman B (2018). Dispensing of antimicrobials in Kenya: a cross-sectional pilot study and its implications. J Res Pharm Pract.

[36] Dela H, Egyir B, Majekodunmi AO, Behene E, Yeboah C, Ackah D, et al. Diarrhoeagenic E. Coli occurrence and antimicrobial resistance of Extended Spectrum Beta-Lactamases isolated from diarrhoea patients attending health facilities in Accra, Ghana. PLoS ONE. 2022;17. 10.1371/journal.pone.0268991. 10.1371/journal.pone.0268991 PMC9135277 35617316

